# Crosstalk Between Oxidative Stress and Epigenetics: Unveiling New Biomarkers in Human Infertility

**DOI:** 10.3390/cells13221846

**Published:** 2024-11-07

**Authors:** Sulagna Dutta, Pallav Sengupta, Filomena Mottola, Sandipan Das, Arif Hussain, Ahmed Ashour, Lucia Rocco, Kadirvel Govindasamy, Israel Maldonado Rosas, Shubhadeep Roychoudhury

**Affiliations:** 1 Basic Medical Sciences Department, College of Medicine, Ajman University, Ajman 346, United Arab Emirates; 2 Centre of Medical and Bio-Allied Health Sciences Research, Ajman University, Ajman 346, United Arab Emirates; 3 Department of Biomedical Sciences, College of Medicine, Gulf Medical University, Ajman 4184, United Arab Emirates; 4 Department of Environmental, Biological and Pharmaceutical Sciences and Technologies, University of Campania Luigi Vanvitelli, 81100 Caserta, Italy; 5 Department of Life Science and Bioinformatics, Assam University, Silchar 788011, India; 6 School of Life Sciences, Manipal Academy of Higher Education (MAHE), Dubai 345050, United Arab Emirates; 7 ICAR-Agricultural Technology Application Research Institute, Guwahati 781017, India; 8 Citmer Reproductive Medicine, Mexico City 11520, Mexico

**Keywords:** epigenetics, DNA modifications, reactive oxygen species, reproductive health, human fertility

## Abstract

The correlation between epigenetic alterations and the pathophysiology of human infertility is progressively being elucidated with the discovery of an increasing number of target genes that exhibit altered expression patterns linked to reproductive abnormalities. Several genes and molecules are emerging as important for the future management of human infertility. In men, microRNAs (miRNAs) like miR-34c, miR-34b, and miR-122 regulate apoptosis, sperm production, and germ cell survival, while other factors, such as miR-449 and sirtuin 1 (SIRT1), influence testicular health, oxidative stress, and mitochondrial function. In women, miR-100-5p, miR-483-5p, and miR-486-5p are linked to ovarian reserve, PCOS, and conditions like endometriosis. Mechanisms such as DNA methylation, histone modification, chromatin restructuring, and the influence of these non-coding RNA (ncRNA) molecules have been identified as potential perturbators of normal spermatogenesis and oogenesis processes. In fact, alteration of these key regulators of epigenetic processes can lead to reproductive disorders such as defective spermatogenesis, failure of oocyte maturation and embryonic development alteration. One of the primary factors contributing to changes in the key epigenetic regulators appear to be oxidative stress, which arises from environmental exposure to toxic substances or unhealthy lifestyle choices. This evidence-based study, retracing the major epigenetic processes, aims to identify and discuss the main epigenetic biomarkers of male and female fertility associated with an oxidative imbalance, providing future perspectives in the diagnosis and management of infertile couples.

## 1. Introduction

Male infertility is the inability of a man to contribute to the conception of a child. This condition can result from various factors that impact the number, quality, functionality, or motility of sperm cells. Although this pathological condition is often linked to genetic irregularities and their interactions, sometimes the exact cause remains unknown. Recent studies on male infertility cases including those of normozoospermia, oligoasthenozoospermia (OAT), and azoospermia report epigenetic aberrations (epimutations) in spermatozoa that can compromise their functionality [1,2,3,4]. These encompass processes such as DNA methylation, modifications to histone proteins, and the action of various types of non-coding RNA (ncRNA), among which the most studied and well known are microRNAs (miRNAs) [5]. Epigenetic modifications include DNA methylation of 5-methylcytosine (5mC), DNA methylation of 5-hydroxymethylcytosine (5hmC), and various modifications of histone proteins like acetylation, methylation, phosphorylation, poly-ADP ribosylation, and ubiquitination. Other categories involve RNA modifications such as 6-methyladenosine (6mA), RNA methylation of 5mC, RNA methylation of 7-methylguanosine (7mG), mRNA cap modification, and RNA methylation of 5hmC [6].

Several genes are known to exhibit epimutations that appear to be involved in the onset of male infertility. Hypermethylation of genes such as *methylenetetrahydrofolate reductase* (*MTHFR*), neurotrophin 3 (*NTF3*), *insulin-like growth factor-2* (*IGF2*), and the *H19* gene for a long ncRNA is indicative of epigenetic changes that can contribute to alterations in semen parameters [7]. In addition, epigenetic regulation of sperm influences the development of the embryo following fertilization [8]. Reports indicate that examining changes in epigenetic markers is critical for minimizing the incidence of abnormal embryo shapes and improving fertility results in assisted reproductive technology (ART) [3].

Like in men, infertility in women is a multifaceted reproductive issue with a variety of causes. These include factors such as the aging of eggs, pathological conditions like polycystic ovary syndrome (PCOS), endometriosis, and repeated miscarriage—all of which are associated with changes in the egg’s epigenetic makeup [9,10,11,12]. Much attention to these factors is needed to understand the molecular basis of female infertility [9]. Similar to sperm cells, oocytes undergo a range of dynamic changes at the epigenetic level, which include DNA methylation, alterations to histones, reshaping of chromosomes, and the presence of ncRNAs. Epigenetic modification during oogenesis directly modulates gene expression and other nuclear processes in the oocyte. It affects oogenesis, resulting in altered chromosome segregation in oocytes, which is associated with infertility, including recurrent miscarriage, idiopathic infertility [10], death of embryos in the uterus [9], and adverse ART outcomes [13]. Studies have shown that aberrant methylation of several genes such as *microsomal epoxide hydrolase* (*1EPHX1*) [14], *follistatin* (*FST*) [15], *hypermethylation of aromatase* (*CYP19A1*) [16], and hypomethylation of *Yes-associated protein 1* (*YAP1*) [17] are associated with PCOS pathogenesis.

**Figure 1:**
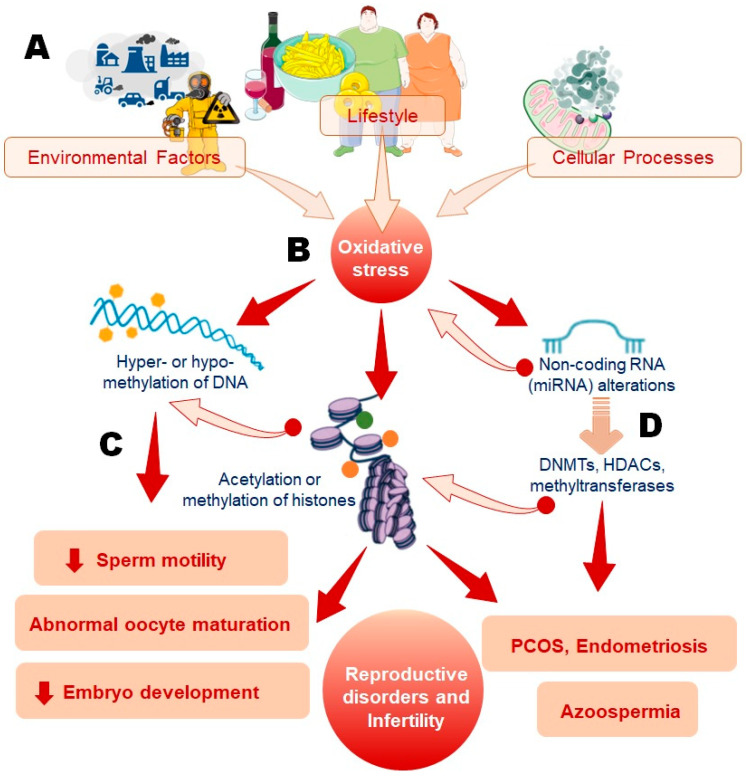
Mechanism of oxidative stress-induced epigenetic alterations and their impact on reproductive health. Environmental factors, lifestyle choices, and cellular processes (**A**) contribute to the generation of oxidative stress by producing various ROS like superoxide anion (O_2_^−^), hydrogen peroxide (H_2_O_2_), and hydroxyl radicals (OH^•^) (**B**). This oxidative stress leads to critical epigenetic modifications, including DNA hyper- or hypomethylation, histone acetylation or methylation, and alterations in non-coding RNAs, particularly microRNAs (**C**). miRNAs interact with epigenetic regulators like DNA methyltransferases (DNMTs), histone deacetylases (HDACs), and methyltransferases, feedback loops where altered miRNA expression can further influence epigenetic processes, reinforcing conditions like oxidative stress (**D**). These epigenetic changes disrupt key reproductive processes, resulting in impaired sperm motility, abnormal oocyte maturation, and compromised embryonic development, ultimately contributing to reproductive disorders such as polycystic ovary syndrome (PCOS), endometriosis, and azoospermia.

Among the factors that can influence the epigenetics of spermatozoa and oocytes, exposure to environmental features can disrupt DNA methylation and induce histone modifications through oxidative stress [18]. It has been reported that superoxide (O_2_^−^), a potent reactive oxygen species (ROS) and precursor of various other free radicals in biological systems, regulates key epigenetic processes, including DNA methylation, histone methylation, and acetylation (Figure 1).

As a radical anion and potent nucleophile, O_2_^−^ can alter epigenetic processes through nucleophilic substitution and free radical abstraction. It can neutralize the positive charges of methyl donors, such as S-adenosyl-L-methionine (SAM) and acetyl-coenzyme A (AcCoA), via nucleophilic reactions, thereby increasing their nucleophilic ability or deprotonating cytosine. Dioxygenase enzymes produce O_2_^−^ through reverse free radical reactions like demethylation and deacetylation, which can then be converted into hydroxyl radicals, leading to the removal of methyl substituents [19]. These processes may help understand how ROS-induced epigenetic modifications contribute to various pathological conditions, including both male and female infertility.

ROS have been shown to alter the methylation patterns of several spermatozoal genes and regulate their expressions to adversely affect spermatogenesis [20]. Studies have revealed that environmental factors are responsible for aberrant epigenetic regulations that bring about deterioration in semen parameters. Previously, the generation of ROS in semen was directly linked to an increase in sperm DNA fragmentation, while inversely relating to sperm DNA methylation [20]. Moreover, DNA methylation has been found to bear negative correlation with DNA fragmentation [20]. Also, infertile men demonstrated both higher DNA fragmentation and ROS levels as compared to fertile men [20]. The data could imply that oxidative stress-triggered DNA damage might amplify unusual global DNA methylation. Furthermore, it has been observed that individuals who undergo antioxidant therapy for a period of three months exhibit a decrease in DNA damage and in ROS levels, along with an upsurge in precise global DNA hypomethylation [20].

The female germline is also vulnerable to oxidative insult [21]. Elevated ROS, particularly O_2_^−^, are involved in impaired chromosome segregation, senescence, and oocyte DNA damage [21,22]. Oxidative stress-induced telomerase enzyme malfunction—leading to point mutation or deletion in the mitochondrial genome, causing reduced ATP production, aberrant meiotic spindle formation, and genomic instability—may finally result in oocyte incompetency [23] and 8-hydroxy-2′-deoxyguanosine (8-OHdG) formation. The oxidized structure of DNA can induce hypomethylation of DNA by interrupting DNA methylation at nearby cytosine residue. Similarly, 5hmC-induced DNA demethylation processes lead to DNA hypomethylation [24].

Since epigenetic modifications can be reversed, and identifying epigenetic biomarkers of infertility could be crucial in enhancing infertility treatment to subsequently achieve successful fertilization [6], this rapidly growing field of research is now increasingly utilizing epigenetic analysis as an indicator of fertility. The purpose of this evidence-based study was to elaborate upon the crucial role of epigenetic regulations in the maturation and functional capacities of spermatozoa and oocytes, with particular attention to the impact of ROS on these processes. Understanding how ROS can cause alterations in epigenetic mechanisms could prove crucial in the treatment of oxidative stress-induced infertility. This article aims to put forth the proposal for one or more specific epigenetic biomarkers to be employed as indicators of ROS impact on the functioning of sperm and oocytes. This could potentially enable the more accurate assessment and subsequent management of fertility issues precipitated by oxidative stress. To accomplish this objective, this evidence-based study has been structured into three main sections: (a) a comprehensive overview of the epigenome of sperm and oocytes, presenting a summary of the existing knowledge; (b) a forecast of oxidative stress-induced epigenetic changes, which could enhance our comprehension of unexplained male and female infertility; and (c) a discussion on potential epigenetic biomarkers of male and female infertility that could serve as measurable indicators for diagnosing fertility complications. Thus, this article seeks to deepen the understanding of the connection between epigenetic alteration, ROS, and human infertility, ultimately contributing to the development of more effective diagnostic and/or treatment strategies.

## 2. Major Epigenetic Processes

The most common and highly characterized epigenetic processes are DNA methylation, histone modifications, chromatin remodeling, and regulation by non-coding RNAs [25].

### 2.1. DNA Methylation

The expression of genes can be dictated by the methylation of cytosine bases, specifically at the five positions, within the context of 5′–C–phosphate–G–3′ (CpG) dinucleotides. CpG islands, which are clusters of these dinucleotides, are located near promoter regions and play a significant role in regulating gene expression [25]. DNA methylation, a vital epigenetic modification, primarily involves the addition of methyl groups to cytosine residues, resulting in the formation of 5mC. This modification significantly contributes to epigenetic regulation and imprinting, as hypermethylation of CpG islands is often associated with gene silencing, while hypomethylation is linked to gene activation [26,27].

The process of DNA methylation is mediated by DNA methyltransferases (DNMTs), which facilitate the transfer of methyl groups from S-adenosylmethionine to the cytosine residues in CpG dinucleotides [26]. Among the DNMTs, DNMT1 is primarily responsible for maintaining established methylation patterns, whereas DNMT3A and DNMT3B are involved in establishing new methylation patterns [28,29,30]. DNMT3L, which lacks its own enzymatic activity, serves as a co-factor for DNMT3A2, enhancing its methylation action [31,32].

The patterns of DNA methylation can vary depending on the species, tissue type, and even the specific cell type. It is believed that the methylation patterns are established during embryonic development and maintained throughout life by DNMTs. However, recent findings indicate that demethylation can occur in mammalian cells to correct improper methylation patterns or activate previously silenced genes [33]. This demethylation process can occur through both active and passive mechanisms, involving the action of ten-eleven translocation (TET) proteins and the AID/APOBEC enzyme family [34].

### 2.2. Histone Modifications

Another essential epigenetic process for proper cell functioning includes the post-translational histone modifications. The N-terminal regions of histone tails are subject to several modifications such as acetylation, methylation, phosphorylation, sumoylation, and ubiquitylation. These combined alterations, along with the genetic data they impart, constitute what is referred to as the histone code. Histone methylation control is facilitated by histone methyltransferases (HMTases), a mechanism that is believed to be involved in the suppression of relevant genes [7]. Histone 3 lysine 4 (H3K4) methylation and histone acetylation are two types of histone post-translational modifications, which are markers of active chromatin structure and normally associated with a lack of DNA methylation [35,36,37]. In contrast, the process of methylation at CpG dinucleotides fosters the formation of a closed chromatin structure. This, in turn, impedes the action of H3K4 methyltransferases, ultimately leading to the suppression of transcription [36,37]. DNA methylation and gene silencing within imprinted genes are linked with other histone modifications, such as methylation of H4K20, H4K27, and H3K8 [35,36,37]. H3K9 methylation is a classic example of gene silencing and is observed in heterochromatin and silenced promoters [38]. Alterations such as methylations on arginine and lysine residues have also been noted to facilitate the activation of genes [39]. Moreover, histone acetylation stimulates transcription, and its regulation is managed by both histone deacetylases (HDACs) and histone acetyl transferases (HATs). Gene expression is activated by HATs and inhibited by HDACs [40]. Bromodomain-containing proteins can specifically recognize the acetylated lysine and augment chromatin remodeling [41]. Gene expression may also be activated via histone phosphorylation on the serine residues [40]. However, H2AX phosphorylation leads to chromosome condensation and gene silencing [42]. Ubiquitylation of lysine residues of histones can aid both gene expression and silencing. For example, ubiquitylation of histone H2A aids gene silencing [43], whereas that of H2B is associated with gene activation [44]. Other modifications of lysines, sumoylation, or attachment of small ubiquitin-related modifier proteins (SUMOs) lead to gene silencing and also inhibit other histone modifications [45]. The organization of chromatin structure at local or global level varies according to the charges carried by the functional groups. The activation or inactivation of chromatin depends on the degree of acetylation or methylation. For instance, variations in the levels of acetylation of H3K27 and H3K9, along with the methylation of H4K20, can influence the transition of euchromatin to heterochromatin states. While it is true that the methylation of H3K27 and H3K9 is often associated with the formation of heterochromatin, the role of acetylation is more complex. Acetylation of these histones generally correlates with active transcription and an open chromatin structure. Therefore, an increase in acetylation may lead to euchromatin states, while a decrease may facilitate the transition to heterochromatin. Indeed, optimum acetylation of H3K9, H3K36, and H3K4 and trimethylation of H3K79 cause the activation of chromatin, whereas a lower degree of acetylation of H3K27 and H3K9 and methylation of H4K20 causes a shift in euchromatin to heterochromatin state [33].

### 2.3. Chromatin Remodeling

Unlike the above-described epigenetic processes, chromatin remodeling does not involve covalent interactions. Chromatin remodeling complexes are ATP-dependent, using ATP hydrolyzing energy to change the structure and/or location of nucleosomes. These changes result in either gene expression or silencing [46]. ATP hydrolysis provides the energy for the change in position and structure of the nucleosome and thus makes genes accessible to transcription factors leading to the expression or silencing of the target gene [46,47,48,49]. The ATPase responsible for chromatin remodeling belongs to the sucrose non-fermentable-2 (SNF-2) family [9]. Studies revealed that the *Brahma-related gene 1* (*BRG-1*) and hBRM have a bromo domain that is susceptible to histone acetylation, leading to chromatin remodeling [47,48,49].

During spermiogenesis, chromatin remodeling involves the replacement of somatic histones with testis-specific variants and subsequent post-translational modifications. These changes, facilitated by enzymes like acetylase and deacetylase, lead to chromatin loosening and DNA strand breaks induced by topoisomerase II beta (Top2β). This process allows for the eviction of histone variants and their replacement with transition proteins (TPs), which are crucial for protamine replacement and sperm DNA condensation in later stages of spermiogenesis [50].

However, it remains to be clarified how transient DNA breaks are repaired. Molecular chaperones may facilitate the replacement of transition proteins with protamines, allowing for dense packaging of DNA. Additionally, it is still unclear how and where histones are degraded and which factor ultimately removes the histones [51], but it has long been established that environmental stressors can influence this process, impairing chromatin remodeling during spermiogenesis [52].

In the neonatal ovaries of mammals, oocytes are naturally stalled in prophase I of meiosis. During the postnatal period, these oocytes remain in prolonged meiotic suspension until puberty, when a rise in luteinizing hormone (LH) prompts the resumption of meiosis [53]. Quiescent oocytes rely on maternal transcripts stored during their maturation and growth phases to complete meiosis and support the initial stages of embryonic development. However, the cellular and molecular mechanisms that coordinate chromatin structure changes and the onset of transcriptional repression remain poorly understood. Histone deacetylases are crucial for chromatin remodeling, and similar to spermiogenesis, disruptions in this process can lead to chromatin alterations that result in abnormalities in chromosomes and meiotic spindles [54].

### 2.4. microRNAs

ncRNAs are molecules that are not translated into proteins but play a crucial role in various biological processes including development, differentiation, cell growth, apoptosis, and pathological processes [55,56]. Mature miRNAs are a family of short single-stranded ncRNA molecules (20–23 nucleotides) capable of regulating post-transcriptional gene silencing through binding to their target mRNAs and degradation or translational repression [48,49]. Over the past decade, miRNAs have also been found to influence complex biological processes such as gametogenesis [57] and are known to be associated with various disorders; for these reasons, they are used for clinical diagnostics and treatment [33]. The biogenesis of miRNAs initiates with the transcription of primary miRNAs (pri-miRNAs), which adopt a hairpin structure, by RNA polymerase II. These pri-miRNAs are processed in the nucleus into precursor miRNAs (pre-miRNAs), which are subsequently transported to the cytoplasm. There, the DICER enzyme, also known as endoribonuclease DICER, further cleaves them into the major and minor complexes, resulting in the formation of mature miRNAs [58,59]. The minor strand is degraded, leaving the mature miRNA (major complex) bound to the Argonaute (AGO) protein, forming the miRNA-induced silencing complex (miRISC). Within this complex, miRNAs function by binding to the 3′ untranslated region (3′ UTR) of mRNAs, leading to mRNA decay or repression of translation [60,61]. A single miRNA can target multiple mRNA molecules, and this interaction between miRNA and mRNA is specific to the stage of development and the type of cell [62]. Dysregulation of certain miRNAs can lead to altered expression of genes involved in gametogenesis, impacting fertility. For instance, miR-23b-3p and miR-320a-3p can modulate the expression of genes critical for sperm cell development, such as 6-phosphofructo-2-kinase/fructose-2,6-biphosphatase 4 (PFKFB4), receptor for hyaluronan-mediated motility (HMMR), and spermatogenesis-associated 6 (SPATA6), serving as biomarkers for spermatogenesis regulation [63].

On the other hand, advanced maternal age is associated with recurrent miscarriage, partly due to the influence of miRNAs. miR-16 regulates vascular endothelial growth factor (VEGF) expression, and high levels impair the proliferation, migration, and formation of human umbilical vein endothelial cells, contributing to recurrent miscarriage [64]. miRNA alterations can affect ovarian reserve, too—miR-100-5p and miR-21-5p levels predict anti-Mullerian hormone (AMH) levels and ovarian reserve status, which influences folliculogenesis, granulosa cell apoptosis, steroidogenesis, and ovulation [65].

In recent years, significant strides have been made in uncovering miRNAs, identifying their targets, and elucidating their functions using both biological and computational approaches. Next-generation sequencing (NGS), including deep sequencing, has been instrumental in the discovery of miRNAs, with resultant sequences archived in databases [66]. Recent advancements in biochemical techniques have further enhanced our ability to identify miRNA targets. For instance, researchers have developed high-throughput sequencing methods like high-throughput sequencing of isolated RNA by cross-linking immunoprecipitation (HITS-CLIP). This technique has been successfully applied to organisms such as mouse brain and *Caenorhabditis elegans*, providing detailed insights into miRNA-mRNA binding sites across both 3′ UTRs and coding regions. Compared to earlier computational methods, HITS-CLIP offers higher specificity and a lower false discovery rate, thereby generating comprehensive genome-wide interaction maps for specific miRNAs [67].

The advent of high-throughput technologies such as microarray, mass spectrometry, and advanced NGS has revolutionized the profiling of various molecules at multiple levels. These technological attributes present new opportunities and challenges in research in this field.

## 3. Epigenetics in the Testes and Spermatogenesis

The epigenetic processes discussed in the above sections create discrete epigenetic patterns in all tissues. Genome-wide analysis conveyed that the testes have unique DNA methylation pattern. Testicular DNA consists of eight times of hypomethylated loci of that of the somatic tissues and most of them are non-CpG islands as well as non-repetitive sequences [68]. As the germ cell advances through the stages of spermatogenesis, the methylation patterns of its genes also alter accordingly, irrespective of their expression patterns [69]. Regulation of gene expression for male reproductive functions are mediated by differentially methylated imprint control regions found between two parental chromosomes [70]. The male germline possesses paternally imprinted genes that are silenced via DNA methylation. There are few genes that have been found to bear paternal imprinting, including the *GTL2, RASGRF1*, and *Igf2/H19* loci [71,72]. The Igf2/H19 locus is reportedly the best-characterized among all the imprinted genes with reciprocal maternal H19 and paternal insulin IGF-2 gene expression [26]. It has been shown that the H19 gene is physically and functionally linked to the *IGF-2* gene [73,74,75]. On the paternal chromosome, the H19 gene and the adjacent differentially methylated region (DMR) are methylated [73]. It seems that the MEST hypermethylation is a marker for decreased motility and abnormal sperm morphology [68]. Abnormal methylation in the MEST locus of paternal sperm may contribute to imprinting disorder in children [76,77]. It has also been shown that hypomethylation of GTL2 (a maternally imprinted gene) plays a critical role in OAT [77]. Hypermethylation of the *MTHFR* gene promoter in sperm is associated with idiopathic male infertility [78]. Also, the imprinting sequences of KCNQ1 overlapping transcript 1 (KCNQ1OT1), small nuclear ribonucleoprotein polypeptide N (SNRPN), and LINE1 (L1) have been implicated in normal chromatin structure [79]. De novo methylation of these elements occurs in male germ cells, resulting in complete methylation in sperm [71,72,73,74,75,76,77,78,79,80]. Spermatogenesis is heavily dependent on post-transcriptional regulatory processes, of which miRNAs are important regulators [81,82]. Testicular expression of miRNA changes between stages of spermatogenesis has been suggested [83,84].

Recent studies have demonstrated that the production of miRNAs in semen, sperm, and testicular tissue and the production patterns of miRNAs tightly correlate with various male diseases and male fertility [55,56]. Furthermore, altered miRNAs have been found in the reproductive cells of infertile patients [85,86]. It was shown that asthenoteratozoospermia (AT) patients have a higher amount of seminal plasma miRNAs than the patients with complete absence of sperm [87].

A number of miRNAs with altered expression have been associated with male infertility pathogenesis, such as Hsa-miR-30a-5p [88], miR-210 [89], miR-10b-3p [90], miR-34b-5p [90], miR-141, miR-429 and miR7-1-3p [91], miR-19b and let-7a [92], hsa-miR-429, hsa-miR-34b*, hsa-miR-34b, hsa-miR-34c-5p, and hsa-miR-122 [93], linking further with non-obstructive azoospermia (NOA). Similarly, hsa-miR-525-3p [94] and miR-151a-5p [95] have been linked with asthenozoospermia, while miR-27a [96] has been linked with asthenoteratozoospermia, whereas hsa-mir-21 and hsa-mir-22 [97] have been associated with oligospermia. The altered miRNA expressions that have been identified in the above-mentioned disorders are summarized in Table 1, with their altered function and target genes and/or signaling pathways.

## 4. Epigenetics in the Ovary and Oogenesis

The perturbation of epigenetic components within the oocyte may precipitate female infertility [9]. Epigenetic processes encompass a plethora of cellular and molecular transformations essential for early embryonic development. The complex interplay of cellular behaviors that dictates zygotic development is reflective of the cellular organization patterns within oocytes [98]. Deviations in methylation patterns and histone modifications may compromise oogenesis, inducing aneuploidy within the fertilized egg and potentially culminating in embryonic mortality. Epigenetic mechanisms implicated in follicular development encompass DNA methylation, histone methylation, and histone acetylation [9]. Oocytes have completely different DNA methylation pattern compared with the DNA methylation pattern observed in sperm or soma [99]. In the oocyte, CpG methylation takes place progressively before it attains the size of 70 µm. A fully mature oocyte accumulates methylation at cytosine residues outside the CpG nucleotide. On the other hand, non-CG methylation represents a significant part of the total methylation in oocytes. Overall, non-CG methylation may appear low by position; however, it is found at significantly higher levels in oocytes compared to other cell types and tissues [100,101].

The advanced age of a mother might negatively impact epigenetic alterations in oocytes. The rate of pregnancy in mammalian models also decreases with advanced age, which may be due to alteration in DNA methylation in oocytes [102,103]. DNA methylation is crucial for imprinting of the genes; failure in imprinting creates congenital anomalies including abnormal growth of placenta, fetal brain and metabolic disorders. Imprinted loci are composed of single or multiple genes, and their expression is controlled by the DNA methylation status of the imprinting control region (ICR) [99]. Hypomethylation of multiple CpG sites of the LH/choriogonadotropin receptor (*LHCGR*) gene results in the elevation of LHCGR transcription levels and is one of the leading causes of anovulation in PCOS patients [103]. With advancing age, alterations also occur in histone acetylation and methylation and in DNMT in oocytes, compromising female fertility and reproductive outcomes [104]. For example, defective deacetylation of H4K12, which promotes elevated levels of ROS in the cytoplasm [105,106], can lead to improper chromosome segregation, potentially resulting in aneuploidy [106]. In the same way, reduced expression of Dnmt1, Dnmt3a, Dnmt3b, and Dnmt3L have been noticed in mammalian old oocytes that alter genome-wide methylation pattern in oocytes and compromise fertility potential [102]. The involvement of altered DNMT1 expression in the onset of female infertility is confirmed by studies on endometriosis. Endometriosis pathogenesis, a medical condition in which the tissue that normally lines the inside of the uterus grows outside of it, leading to fertility problems, is mainly regulated by hypoxia that down-regulates DNMT1 through miR-148a and causes global hypomethylation, whereas inflammation triggers a rise in DNMT3a loci-specific hypermethylation. Both hypoxia and inflammation regulate methylation of DNA via miRNAs [107]. Scientific reports have suggested that aberrant methylation at promoters and/or introns [108] of different genes such as aromatase (CYP19) [109], steroidogenic acute regulatory protein (StAR) [110], cyclo-oxygenase (COX-2) [111], estrogen receptor (ER) b12 [112], and steroidogenic factor (SF)-1 [35] can impair reproductive functions. Recent studies have demonstrated that a number of altered miRNAs’ expressions are linked with female reproductive disorders. Altered expression of miR-320a [113], miR-93 [114,115], miR-132 [116], miR-222-3p [117], miR-126-5p and miR-29a-5p [118], miR-592 [119], and miR-21 [120] have been associated with PCOS pathogenesis. Similarly, miRNAs such as miR-29c [121], miR-194-3p [122], miR-191 [123], miR-199a-5p [124], and miR-20a [125] have been linked with development of endometriosis pathogenesis. Ten altered miRNAs have been associated with recurrent pregnancy loss, namely, hsa-miR-221-3p, has-let-7e, hsamiR-16, hsa-miR-519d, hsa-miR-410, hsa-miR-184, hsa-miR-21, hsa-miR-125, hsa-let-7a and hsa-let-7d, and miR-126 [126].

The altered miRNA expressions identified in the above-mentioned female disorders are summarized in Table 2, with their altered functions and target genes and/or signaling pathways.

## 5. Oxidative Stress and Critical Epigenetic Changes in Male Infertility

One of the most evident reasons for alterations in the sperm epigenome is its interaction with both external and internal ROS. Furthermore, environmental factors, biological characteristics, aging, illness, obesity, and infertility also play significant roles in these changes.

Evidence suggests that the environmental influences cause not only epigenetic modifications in the exposed organism but can also produce endogenous ROS through multiple cellular mechanisms such as NADPH oxidase (NOX) complexes in cell membranes, mitochondria, peroxisomes, and endoplasmic reticulum [19]. Evidence for the role of ROS in modulating the DNA methylome has emerged from studies on cancer cells. These cells, often under oxidative stress, exhibit significant changes in their methylation status [30]. Nevertheless, in non-cancerous tissues too, ROS may induce alteration in the methylome [127,128,129,130].

A recent study has reported that higher oxidative stress level leads to hypermethylation of repetitive elements like LINE1 [131]. Several mechanisms may link increased ROS levels to changes in DNA methylation patterns. For example, ROS-induced DNA damage can modulate DNMT activity and alter the binding of DNMT-containing complexes [132]. Yet another study has demonstrated a noteworthy augmentation in the methylation of the *MLH1* gene promoter in patients suffering from oligozoospermia, when compared with controls who had normal sperm count. This pattern has been found to be positively correlated with heightened levels of ROS in semen. *MLH1* gene plays a critical role in the DNA mismatch repair process and in the crossing over during meiosis, making it a significant factor in male fertility issues [133]. Oxidative stress may affect the sperm chromatin structure and the epigenetic regulation in at least two relevant manners including the protamine content and the epigenetic markers [134]. Therefore, it may induce DNA methylation alteration, chromosome instability, DNA fragmentation, and sperm aneuploidy [135]. Studies have demonstrated the association of semen ROS with abnormalities in transition of sperm histone. Protamine in mature sperm can alter DNA neutrality, prevent RNA synthesis, and restrict the expression of sperm genes [136,137,138,139,140,141,142,143,144,145,146,147,148,149,150,151,152,153,154,155,156,157,158,159,160,161,162,163,164,165,166,167,168,169,170,171,172,173,174,175,176,177,178]. Abnormalities in histone-to-protamine transition may hinder sperm DNA stability and interfere with normal depolymerization occurring in the sperm nuclei, thus deteriorating the fertilizing potential of sperm, as well as embryonic development [139,140]. High ROS levels, via interference in sperm epigenetic stability, turns out to be one of the most sorted risk factors in unexplained miscarriages and failure in embryo development. Detailed mechanisms of ROS-induced abnormalities in histone transition have not been documented yet.

To our knowledge, there is no study that has specifically investigated the effect of oxidative stress on the alteration of DNA methylation in paternally and maternally imprinted genes like H19/IGF2 and PEG1/MEST loci. In germ cell recombination process, homologous chromosome hotspots remain hypomethylated and decondensed, and full chromatins are condensed to facilitate the heterochromatin state [140,141]. Additionally, it has been reported that the guanosines in telomeres, which are repeated TTAGGG sequences enriched with guanosine and several thousand base pairs long, are prime targets for oxidative damage that structurally persists and cannot be repaired. Besides chromatin methylation, telomere oxidation also contributes to gamete aneuploidy [142].

## 6. Oxidative Stress and Critical Epigenetic Changes in Female Infertility

Optimum levels of free radicals are crucial for the processes of cell communication, correct operations in the formation of ovarian follicles, egg cell maturation, degradation of *corpora lutea*, and the embedding of embryos and their subsequent growth [143,144,145]. ROS result from exogenous oxidizing agents that include hypoxia, Hb, heme, and heavy metals or from spontaneous reactions carried out in mitochondria or in metabolic process. Major ROS include superoxide anion radical, hydrogen peroxide, and hydroxyl radical, which play an important role in regulating cell survival, senescence, and aging through a variety of mechanisms [146]. In endometriosis pathogenesis, oxidative stress plays a significant role. Oxidative stress generates from hemoglobin (Hb)-, heme-, and ion accumulation-induced ROS due to repeated hemorrhage [147]. Oxidative stress can alter epigenetic processes by removing DNA and histone methylation marks. ROS convert Fe^2+^ to Fe^3+^, thereby inhibiting Jumonji family histone demethylase activity and enhancing DNMT activity [146,147,148]. On the other hand, ROS may cause site-specific alteration in the methylation pattern through regulating the expression of DNMTs. Hydrogen peroxide (H_2_O_2_) may induce hypermethylation at the target site by recruiting DNMTs [149]. Uncontrolled methylation in endometriosis can cause activation or suppression of target genes involved in hormonal regulation, cell cycle, cell adhesion, and tumor suppression activity. Oxidative stress-induced DNA hypermethylation leads to defective endometrium maturation [147]. In contrast, excess ooplasmic ROS has been linked to hyperacetylation of histone H4 at lysine 12 in mammalian PCOS ovaries. Such altered epigenetic modification impairs the maturation of oocytes, too [149]. As mentioned earlier, methylation takes place at CpG islands, and guanine (G) is most sensitive to oxidative insult, leading to the formation of 8-ox-deoxyguanosine. Similarly, oxidative byproducts of cytosine (C) include 5-OH C, 5,6- diOH C, and C glycol [150]. Due to base oxidation alterations to DNA site interactions and transcription factors, aberrant heritable epigenetic changes may occur. An elevated amount of 5HmC—a byproduct of C oxidation—contributes to alteration of epigenetic process via disruptive DNA demethylation [32].

## 7. Male Infertility Candidate Epigenetic Biomarkers

In the context of fertility, a biomarker provides information about reproductive health or the ability to conceive [63]. miRNAs can serve as biomarkers of male fertility or infertility due to their regulatory roles in gene expression and their involvement in various reproductive processes. Changes in the expression profile of miRNA in patients experiencing various forms of spermatogenic dysfunction could potentially lead to the development of novel biomarkers for diagnostic use [56,85,86,87,91,93]. In germ cells, miR-34c is produced in the late stages of meiosis (pachytene spermatocytes and round spermatids) [83,84,151]. It plays a vital role in apoptosis, p53-mediated cell death, and the control of cell cycle, especially the first cell division via modulation of Bcl-2 expression [83,84,151]. miR-34c expression has been reported to be down-regulated in the seminal plasma of azoospermia patients and up-regulated in the seminal plasma of AT patients [87]. miR-34b has reportedly been down-regulated in both OA and azoospermia patients and highly expressed in normal adult testis [152]. The putative target gene regulated by miR-34b and miR-34c is notch gene homologue 1 (NOTCH1), which is highly expressed in mature testis and is requisite for the differentiation and survival of germ cells [83,153]. miR-34b* has been observed to be lowered in individuals diagnosed with NOA as well as in men having subfertility issues associated with OAT [56,85]. miR-122 has been linked with reproductive health issues, infections, inflammation, cell death, abnormal testis growth, and sperm production [154,155]. It helps decrease the production of transition protein 2 (TNP2) by targeting TNP2 mRNA’s UTR [156]. Structurally similar miR-449 is a possible indicator for sperm production and testicular health [151,157,158,159]. It is expected to target genes involved in apoptosis (caspase-2 and BCL2), transcription (NOTCH1), and hormone regulation (inhibin βB) [160,161]. E2F transcription factor 1 (E2F1) positively influences miR-449 expression [162,163]. If E2F1 is lacking, sperm cell proliferation declines significantly, leading to testicular shrinkage [164]. miR449 promotes cell death independently of p53, suggesting that imbalances in these miRNAs could lead to increased cell death [85,165,166]. miRNAs that seem to play a crucial role in oxidative stress and mitochondrial dysfunction have been reported previously [167]. Excessive ROS or aging can also decrease sirtuin 1-targeting (SIRT1) miRNA expression [168]. SIRT1 activation can improve oxidative stress response and promote eNOS-derived NO bioavailability and mitochondrial biogenesis [169]. Notably, SIRT1 is also a target of miR-34, which significantly increases in the pro-apoptotic pathway [169,170], as mitochondrial injury has been shown to correlate with oxidative stress and specific miRNAs can affect mitochondrial integrity. miR-16 is a regulator of ATP levels and down-regulates the expression of the ADP ribosylation factor-like 2 (Arl2) mRNA as a common protein target [135]. In summary, most of these miRNAs likely have a common function of limiting sperm production and promoting cell death.

## 8. Female Infertility Candidate Epigenetic Biomarkers

miRNAs are differentially expressed in different types of reproductive disorders, and a single miRNA may target hundreds of genes and thus be involved in the complex molecular network of female reproductive health [171]. miR-100-5p expression profiling may help in the diagnosis of infertile female patients. It may serve as a diagnostic tool for identifying the ovarian reserve in the female experiencing fertility related issues [172]. Additionally, miR-100-5p overexpression has been noted in endometriosis pathophysiology [173] while lower expression of miR-100-5p was observed in the case of ectopic pregnancy [174]. In patients with PCOS, there has been a noticeable decrease in the levels of miR-483-5p and miR-486-5p in the cumulus cells of metaphase II oocytes. miR-483-5p has a significant role in insulin resistance, and the reduced presence of miR-486-5p has been linked to an increase in PTEN expression within cumulus cells. This overexpression of PTEN is considered one of the potential causes of PCOS [175]. Expression profiling of both these miRNAs can be a potential marker in the evaluation of PCOS. During follicular development, miR-320 maintains steroidogenesis by targeting E2F1 and SF-1, and overexpression of miR-320 in granulosa cells in PCOS pathogenesis has been associated with estrogen deficiency via targeting RUNX2 [176]. In mammalian models, miR-28-5p has reportedly reduced PCOS pathogenesis by targeting the 3′-UTR of PROK1, which has involvement in the PI3K/AKT/mTOR signaling pathways, indicating the miR-28-5p/PROK1 axis as a potential target in PCOS treatment [177]. Screening of a panel of five miRNAs—miR-17-5p, miR-20a-5p, miR-143-3p, miR-199a-3p, and let-7b-5p—as an epigenetic signature with high sensitivity (0.96) and specificity (0.79), similar to laparoscopy, has been suggested in order to distinguish normal healthy females from endometriosis patients [178]. The critical function of miRNAs in sustaining female fertility cannot be overstated, and any modification(s) to these miRNAs can have negative implications for fertility capabilities. For example, studying miR-100-5P, miR-483-5p, and miR-486-5p may shed light on the complex molecular processes behind changes in female fertility.

Recent literature supports the hypothesis that miRNAs and oxidative stress are linked through a vicious cycle and that oxidative stress regulates the biosynthesis of numerous miRNAs. In contrast, aberrant expression of miRNAs leads to the development of oxidative stress by facilitating the generation of ROS or reducing endogenous antioxidant potential [179,180,181,182]. It has been shown that up-regulation of miR-200c impairs the regulatory loop among SIRT1, FOXO1, and eNOS and elevates ROS production and reduces cellular NO level, leading to endothelial cell growth disruption. This event promotes ROS production and decreases NO, contributing to endothelial dysfunction and apoptosis [183]. In the case of endometriosis, miR-21, miR-23a-3p, and miR-9-5p have been linked to the regulation of ROS production. Both miRNAs and oxidative stress may generate separate effects such as increased invasiveness, proliferation, and apoptosis, leading to endometriosis [184,185].

## 9. Conclusions and Future Perspective

**Figure 2:**
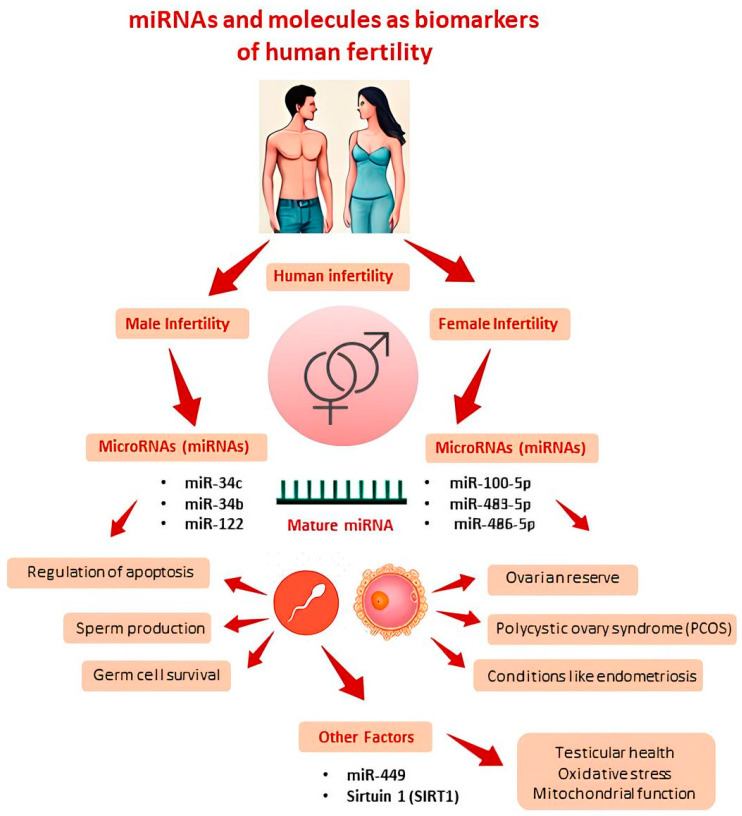
Key candidate microRNAs and other molecules as biomarkers of human fertility. miRNAs are involved in the regulation of gene expression in key processes of male reproduction such as sperm production, apoptosis, cell survival, and oxidative processes. In women, they are associated with ovarian reserve and conditions like polycystic ovary syndrome (PCOS) and endometriosis. The analysis of their expression profiles can provide valuable information for the diagnosis and management of fertility issues.

Epigenetic processes significantly maintain male and female fertility, with emerging data implicating epigenetic alterations in idiopathic infertility. This involves DNA methylation, histone modifications, and miRNA-mediated post-transcriptional gene regulation affecting physiological and pathological functions, including spermatogenesis, oogenesis, and associated reproductive disorders. An exhaustive literature survey underscored aberrant miRNA concentrations in human semen during NOA, oligospermia, and asthenozoospermia, and in follicular fluid in clinical conditions like PCOS, endometriosis, and recurrent pregnancy loss, revealing that specific miRNAs are emerging as crucial biomarkers for the diagnosis and management of infertility in both men and women (Figure 2).

In men, miRNAs such as miR-34c, miR-34b, miR-122, and miR-449, which are involved in key processes related to sperm production, germ cell survival, and testicular health, appear to be the main candidates. For instance, miR-34c and miR-34b regulate apoptosis and cell cycle progression, which are vital for maintaining normal spermatogenesis. Meanwhile, miR-122 is associated with reproductive health issues such as infections and abnormalities in testicular growth. The role of these miRNAs in regulating oxidative stress and mitochondrial function further underscores their potential in the diagnosis and development of therapeutic strategies for male infertility.

In women, miRNAs such as miR-100-5p, miR-483-5p, and miR-486-5p are essential for assessing ovarian reserve and are implicated in conditions like PCOS and endometriosis. Dysregulation of these miRNAs can impact hormonal regulation, insulin resistance, and oxidative stress, contributing to fertility issues. Additionally, panels of miRNAs, including those like miR-17-5p and let-7b-5p, are being studied for their potential to provide accurate diagnoses for conditions such as endometriosis, thus assisting healthcare providers in making informed therapeutic decisions. These findings validate the potential of ncRNAs as diagnostic and prognostic biomarkers, augmenting the therapeutic management of infertile couples and addressing unexplained infertility issues.

The precise identification of epigenetic modifications facilitates comprehensive infertility diagnosis, superseding traditional methods. As epigenetic modifications are potentially reversible, unlike genetic mutations, they propel scientific efforts towards novel therapeutics to reinstate proper epigenetic expression.

While considerable progress has been achieved in epigenetic drug development for pathological conditions, such as cancer, we still need to tread a significant path for fertility treatment. The aim in infertility management should be pre-empting epigenetic alterations. A robust association exists between oxidative stress and certain epigenetic changes, with excess ROS leading to alterations in DNA methylation, acetylation patterns, and the biosynthesis of fertility-related miRNAs. Thus, combating oxidative stress may offer an immediate, simpler strategy to prevent epigenetic disorders inducing reproductive failure.

Multiple studies attest to the positive impact of antioxidants on seminal parameters and female reproductive functions [186,187,188,189], thereby suggesting their potential role in modulating ROS-dependent epigenetic mechanisms causing infertility.

Future research should focus on the epigenomic evaluation of post-antioxidant therapy in infertile individuals to elucidate the specific molecules’ influence on the reproductive epigenome. Current evidence supporting antioxidants’ benefits on fertility suggests their potential as a basis for developing efficacious infertility therapies.

This comprehensive study provides invaluable insights into the intricate interplay of epigenetic processes in fertility and infertility, emphasizing the need for further investigation and development in this area. The revelations contained herein are of pivotal importance to the field of reproductive medicine, heralding new avenues for diagnosing, preventing, and treating fertility issues.

## Figures and Tables

**Table 1:** MicroRNAs (miRNAs) associated with different male reproductive disorders.

miRNA	Sample Type/Tissue Type	Expression Level	Target Gene(s)/Signal Cascade	Clinical Condition	Altered Biological Function(s)	Reference
*Hsa-miR-30a-5p*	Testicular tissue	Up-regulation	*KDM3A*	Non-obstructive azoospermia	Testicular malfunction or inadequate gonadotropin production	[88]
miR-210	Testicular tissue	Up-regulation	*IGF2*	Non-obstructive azoospermia	Testicular malfunction or inadequate gonadotropin production	[89]
miR-10b-3p	Testicular tissue	Up-regulation	-	Non-obstructive azoospermia	Testicular malfunction or inadequate gonadotropin production	[90]
miR-34b-5p	Testicular tissue	Down-regulation	-	Non-obstructive azoospermia	Testicular malfunction or inadequate gonadotropin production	[90]
Hsa-miR-525-3p	Sperm cells	Down-regulation	*SEMG1*	Asthenozoospermia	Reduced sperm motility	[94]
miR-141, miR-429, miR7-1-3p	Sperm cells	Up-regulation	-	Non-obstructive azoospermia	Testicular malfunction or inadequate gonadotropin production	[91]
miR-19b let-7a	Seminal plasma	Up-regulation	-	Non-obstructive azoospermia/oligospermia	Spermatogenesis failure	[92]
hsa-mir-21 and, hsa-mir-22	Sperm cells	Up-regulation	*ERβ*	Oligospermia	ERβ expression significantly low	[97]
miR-27a	Semen	Up-regulation	*CRISP2*	Asthenoteratozoospermia	Impairment of sperm motility, acrosome reaction and gamete fusion	[96]
miR-151a-5p	Semen	Up-regulation	*CYTB*	Asthenozoospermia	Impairment of sperm motility by reducing ATP production	[95]
hsa-miR-429	Semen	Up-regulation	-	Subfertile and non-obstructive azoospermia	Impairment of sperm production	[55]
hsa-miR-34b, hsa-miR-34c-5p, and hsa-miR-122	Semen	Down-regulation	-	Subfertile and non-obstructive azoospermia	Impairment of sperm production	[55]

**Table 2:** MicroRNAs (miRNAs) associated with different female reproductive disorders.

miRNA	Sample Type/Tissue Type	Expression Level	Target Gene(s)/ Signal Cascade	Clinical Condition	Altered Biological Function(s)	Reference
miR-320a	Cumulus granulosa cells	Down regulation	*IGF1*	PCOS	Impaired steroidogenesis	[113]
miR-93	Ovarian granulosa cells	Up-regulation	*CDKN1A*	PCOS	Promotion cell proliferation and progression of G1 to S transition	[114]
miR-93	Adipose tissue	Up-regulation	*GLUT4*	PCOS	Insulin resistance	[115]
miR-132	Follicular fluid	Up-regulation	*Foxa1*	PCOS	Inhibition granulosa cell viability	[116]
miR-222-3p	Serum	Up-regulation	*PGC-1α*	PCOS	Increased risk of cardiovascular complication and diabetes	[117]
miR-126-5p and miR-29a-5p	Ovarian granulosa cells	Down-regulation	miR-126-5p, miR-29a-5p/klotho/insulin-IGF-1, Wnt and Akt signaling pathway	PCOS	Apoptosis in granulosa cell and enhance PCOS progression	[118]
miR-592	Serum	Down-regulation	*LHCGR*	PCOS	Inhibition of cell viability and transition of phase G1 to phase S.	[119]
miRNA-21	Serum	Up-regulation	*LATS1*	PCOS	Promotion PCOS progression	[120]
miR-29c	Endometrium tissue	Down-regulation	*c-Jun*	Endometriosis	Suppression of cell proliferation and promotion apoptosis	[121]
miR-194-3p	Endometrial stromal cells	Up-regulation	-	Endometriosis	Decrease progesterone receptor expression	[122]
miR-191	Serum	Up-regulation	*TIMP3*	Endometriosis	Increased cell proliferation	[123]
miR-199a-5p	Serum	Down-regulation	*SMAD4*	Endometriosis	Promotion cell proliferation, motility and angiogenesis leading to endometriosis progression	[124]
miR-20a	Ovarian tissue	Up-regulation	*NTN4*	Endometriosis	Impairment of cell cycle pathway and promotion endometriosis through angiogenic response	[125]
Hsa-miR-221-3p	Blood plasma	Up-regulation	-	Recurrent pregnancy loss	-	[126]
Has-let-7e	Blood plasma	Up-regulation	-	Recurrent pregnancy loss	-	[126]
HsamiR-16	Blood plasma	Up-regulation	-	Recurrent pregnancy loss	-	[126]
Hsa-miR-519d	Blood plasma	Up-regulation	-	Recurrent pregnancy loss	-	[126]
Hsa-miR-410	Blood plasma	Up-regulation	-	Recurrent pregnancy loss	-	[126]
Hsa-miR-184	Blood plasma	Up-regulation	-	Recurrent pregnancy loss	-	[126]
Hsa-miR-21	Blood plasma	Down-regulation	-	Recurrent pregnancy loss	-	[126]
Hsa-miR-125	Blood plasma	Down-regulation	-	Recurrent pregnancy loss	-	[126]
Hsalet-7a	Blood plasma	Down-regulation	-	Recurrent pregnancy loss	-	[126]
Hsa-let-7d	Blood plasma	Down-regulation	-	Recurrent pregnancy loss	-	[126]
miR-126	Blood plasma	Down-regulation	-	Recurrent pregnancy loss	Involved in angiogenesis through promoting VEGF expression	[11]

## Data Availability

Not applicable.
